# Does opportunistic testing bias cognitive performance in primates? Learning from drop-outs

**DOI:** 10.1371/journal.pone.0213727

**Published:** 2019-03-20

**Authors:** Michèle N. Schubiger, Alexandra Kissling, Judith M. Burkart

**Affiliations:** 1 Department of Anthropology, Evolutionary Cognition Group, University of Zurich, Zurich, Switzerland; 2 School of Social and Health Sciences, Division of Psychology, Abertay University, Dundee, Scotland, United Kingdom; University of New England, Australia, AUSTRALIA

## Abstract

Dropouts are a common issue in cognitive tests with non-human primates. One main reason for dropouts is that researchers often face a trade-off between obtaining a sufficiently large sample size and logistic restrictions, such as limited access to testing facilities. The commonly-used opportunistic testing approach deals with this trade-off by only testing those individuals who readily participate and complete the cognitive tasks within a given time frame. All other individuals are excluded from further testing and data analysis. However, it is unknown if this approach merely excludes subjects who are not consistently motivated to participate, or if these dropouts systematically differ in cognitive ability. If the latter holds, the selection bias resulting from opportunistic testing would systematically affect performance scores and thus comparisons between individuals and species. We assessed the potential effects of opportunistic testing on cognitive performance in common marmosets *(Callithrix jacchus)* and squirrel monkeys *(Saimiri sciureus)* with a test battery consisting of six cognitive tests: two inhibition tasks (Detour Reaching and A-not-B), one cognitive flexibility task (Reversal Learning), one quantity discrimination task, and two memory tasks. Importantly, we used a full testing approach in which subjects were given as much time as they required to complete each task. For each task, we then compared the performance of subjects who completed the task within the expected number of testing days with those subjects who needed more testing time. We found that the two groups did not differ in task performance, and therefore opportunistic testing would have been justified without risking biased results. If our findings generalise to other species, maximising sample sizes by only testing consistently motivated subjects will be a valid alternative whenever full testing is not feasible.

## Introduction

Cognitive performance in animals can be assessed as pure proof of principle, i.e. to test whether a specific ability can be found in a given species. However, cognitive performance is increasingly assessed also for comparative purposes, both between and within species. At the inter-specific level, two or more species are tested with the same cognitive task(s) in order to explore species differences and similarities in cognitive abilities. These differences are usually hypothesised to emerge because the species have faced different selection pressures in their evolutionary past, such as challenges posed by their social or ecological environment. Examples of such studies suggest that enhanced inhibitory control is found in species with a fission-fusion social group structure [1] or a feeding ecology that requires more patience [2], that more tolerant species show increased socio-cognitive performance [3], and that species relying on a diet rich in fruit [4] or food caching species [5] show enhanced spatial memory. Encountering specific social and ecological challenges can thus, over evolutionary time, lead to domain-specific cognitive adaptations.

Cognitive comparisons can also focus on the individual level, allowing researchers to investigate differences and similarities between individuals of the same species. Examples include studies exploring the effects of aging [6–8], sex differences (e.g. [9, 10]), or links between cognitive performance and personality (e.g. [11, 12]). Finally, individual differences can be compared across a variety of cognitive tasks. If cognitive abilities co-vary across individuals of the same species, this is consistent with the notion of domain-general cognition rather than domain-specific cognitive adaptations, and thus general intelligence which facilitates solving a wide range of problems, particularly novel ones (see [13] for a review).

Whenever the goal is to compare cognitive performance across species and individuals, it is crucial to take into account that an individual’s performance in a test is also affected by factors that are not primarily cognitive in nature, such as emotion, motivation and health. We may thus risk measuring individual differences in such non-cognitive factors rather than cognitive ability per se and therefore the performance scores would be biased or even meaningless. The same is true if individuals are selected for inclusion in a cognitive study based on such non-cognitive factors.

In the last decade, researchers have started to systematically re-address the risk that cognitive tests may inadvertently measure individual (and species) differences in non-cognitive factors rather than true differences in the cognitive abilities. A number of experimental studies in non-human primates has systematically assessed both external (testing-related) and internal (subject-related) factors to determine how they affect the subjects’ cognitive performance (Table 1). Examples for internal factors that affect cognitive performance in physical cognition tasks include individual differences in the subjects’ psychological (e.g. temperament and personality structure [14–16], and emotional reactivity [10]) or physical predispositions (e.g., hand preference [17–18]), and individual differences in the subjects’ ontogenetic experience with the social environment (e.g. rearing conditions [19], level of contact with humans [20] and conspecifics [21]).

**Table 1:** Non-cognitive internal (subject-related) and external (testing-related) factors potentially affecting cognitive performance (in physical cognition tasks). Increase (↑), decrease (↓) or no effect (=) on performance.

Factor	Cognitive task(s)/skills	Effect on performance?	Species	Reference
Trait anxiety	Reversal learning	Performance ↓ in subjects with trait anxiety	Long-tailed macaques (*Macaca fasciularis*)	Toxopeius et al., 2005
Temperament	Physical cognition	Performance ↑ in bolder subjects	Chimpanzees (*Pan troglodytes*) & orangutans (*Pongo pygmaeus*)	Herrmann et al., 2007
Personality	Training	Performance ↑ in subjects with high openness & low assertiveness scores	Brown capuchins (*Sapajus apella*)	Morton et al., 2013
Emotional reactivity	Object permanence	Participation ↓ but performance = in subjects with elevated arousal levels during testing	Common marmosets (*Callithrix jacchus*)	Schubiger et al., 2015
Hand preference	Problem solving	Exploration ↑ in subjects with a right-hand preference but performance = in right-handed and left-handed marmosets	Common marmosets (*Callithrix jacchus*)	Cameron & Rogers, 1999
	Cognitive bias	Negative cognitive bias (↓ exploration of an ambiguous test stimulus) in left-handed but not right-handed subjects		Gordon & Rogers, 2015
Rearing conditions	Repertoire of learned cognitive skills	1. Skill repertoire ↑ in mother-reared individuals 2. Skill repertoire ↓ in orphaned individuals	Various primate species	Reviewed in: van Schaik & Burkart, 2011
		Set of skills & learning speed ↑ in enculturated individuals; even beyond a species’ natural repertoire	Great apes	
Degree of orientation towards humans	Problem-solving	Performance ↑ in subjects with high HOI (Human-Orientation Index)	Sumatran (*Pongo abelii*) & Bornean orangutans (*Pongo pygmaeus*)	Damerius, Forss et al., 2017
Human care & social housing with conspecifics	Inhibitory control, reversal learning, problem solving, causal reasoning	Performance ↑ with curiosity & exploration (“curious response-and-exploration style”)		Damerius et al., 2017
Task format	Quantity discrimination	Performance ↑ 1. with inedible test stimuli 2. with edible test stimuli if reward items differ in food type	Olive baboons (*Papio anubis*) & long-tailed macaques (*Macaca fascicularis*)	Schmitt et al., 2011
		Performance ↑ with edible test stimuli	Brown capuchins (*Cebus sapajus apella*)	Gazes et al., 2017
		Performance ↑ with rewards of higher value	Brown capuchins (*Cebus sapajus apella*) & common squirrel monkeys (*Saimiri sciureus*)	
	Memory	1. Performance ↑ with more choice options (9 instead of 2) 2. Performance ↓ with delay duration (in line with forgetting curve)	Common squirrel monkeys (*Saimiri sciureus*) & common marmosets (*Callithrix jacchus*)	Schubiger et al., 2016^1^
	Visual object discrimination	Performance ↑ when tactile exploration of the objects is possible	Capuchin monkeys *(Sapajus spp*.*)*	Carducci et al., 2018
Opportunistic testing	Inhibitory control & memory	Performance = when excluding subjects who take longer to complete all test trials	Common marmosets (*Callitrix jacchus*) & squirrel monkeys (*Saimiri sciureus*)	This study
	Quantity discrimination & reversal learning		Common marmosets (*Callithrix jacchus*)	

^1^see [26] for a similar positive effect of more choice options in an object choice task)

Examples of external non-cognitive factors that have been addressed so far include aspects of the test design, setup and procedure such as how the test apparatus and test stimuli are presented to the subject (e.g. task format [22–25], also see [26] for a similar effect in a social cognition task), and how subjects are rewarded when they pass a test trial (e.g. reward type [23]).

Despite these recent efforts to assess potential biases on cognitive performance, one important aspect of comparative testing has largely been neglected. Comparative testing requires a sufficiently large sample size to make valid inferences. Researchers therefore should test a sufficient number of individuals to reach adequate statistical power, often under logistic restrictions such as limited access to testing facilities. One way to deal with these constraints is opportunistic testing, i.e. only testing those individuals who readily participate and respond to the experimental tasks and drop those who respond too slowly or to erratically. By only including the readily participating individuals, researchers can maximise sample size if enough potential subjects are available, but only limited testing time. Some subjects may be more reluctant to participate because of non-cognitive attributes such as a tendency to react to the test situation with elevated emotional arousal. Those subjects may no longer be motivated to participate in a cognitive test after a relatively low number of completed trials or get easily distracted during testing (as found in marmosets, particularly males [10]). Other subjects, however, may be reluctant to participate because their cognitive skills do not allow them to pass the task and earn rewards. If so, their exclusion would be detrimental to any meaningful comparison of cognitive ability.

An open question is therefore whether opportunistic testing, here defined as using strict stop criteria to restrict total testing time, results in a selection bias because the subjects included in the study differ in cognitive ability from a random sample of the population.

The aim of the present study was to address this question in two New World primate species, common marmosets *(Callithrix jacchus)* and squirrel monkeys *(Saimiri sciureus)*. The subjects were tested with a cognitive test battery consisting of five commonly used paradigms: Detour Reaching, A-not-B, Quantity Discrimination, Reversal Learning, and a two-choice Memory task (largely adapted after an existing test battery for New World monkeys [27]), as well as a nine-choice Memory task [24]). Importantly, in contrast to opportunistic testing, we gave each subject as much time as it needed to complete each task at its own pace (but within a reasonable time frame that ensured subjects were only excluded after permanent motivation loss). We then compared, for each cognitive task, the performance of subjects who had completed the task within the expected time frame (i.e. a predefined number of days or trials to complete the task) to the performance of those subjects who needed longer. If cognitive ability had an effect on how long an individual took to complete a cognitive test, then subjects who needed longer to complete a test should either perform more poorly or better than those subjects who completed the test in the expected time frame. In this case, opportunistic testing would indeed bias the results and would need to be abandoned in future studies. If there was no difference between the two groups, however, the commonly used opportunistic testing approach that excludes some subjects would be equally justified without risking a selection bias in the results.

## Materials and methods

### Study sample

As summarized in Table 2, twenty-seven adult common marmosets *(Callithrix jacchus)* from six family groups and eight adult common squirrel monkeys *(Saimiri sciureus)* from two bachelor groups participated in this study. The initial study sample consisted of the fifteen marmosets (seven females and eight males) in the upper section of the table who were tested with the whole test battery of subtests 1–5 (hereafter tasks). Kitty (female) who became Kantor’s partner later in the study (after he had to be separated from his brothers Kaliper and Kapi) was tested with task 5. The remaining eleven marmosets (five females and six males) were only tested with both memory tasks (5 and 6) when we assessed in a parallel study whether memory task 5 truly measured the ability to remember the location of a reward [24]. The squirrel monkeys (eight males) were only tested with tasks 1, 2, 5 and 6 because their temporal availability for testing was restricted. The study subjects had previously participated in some cognitive experiments but we randomly chose those subjects to include in our study who were available because they were not participating in other studies of cognitive or behavioural nature at the time. We tested all individuals of both squirrel monkey groups (the only two groups of this species that were housed at the Primate Station) and all adult individuals of the available marmoset family groups. MNS conducted all experiments with the exception of the squirrel monkeys and the second marmoset sample in the memory tasks whom were tested by AK.

**Table 2:** Individual subjects by species and family group, their sex (female/male), mean age (in years) during testing, time needed to complete and performance scores in each task. A performance score value in regular font indicates that the subject completed the cognitive task and its performance could be fully analysed whereas a performance score value in italics indicates that the subject did not complete the full task but its performance could be analysed for parts of the task. A dash (-) indicates that the subject participated in such a small number of trials that its performance could not be analysed and an empty cell indicates that a subject refused to participate at all. Grey cells indicate that a subject was not tested with a given task.

ID	Individual	Species	Sex	Age	1. Detour Reaching			2. A-not-B		3. Numerical Discrimination		4. Reversal Learning		5. Memory 1				6. Memory 2	
					Days	Correct inhibition trials at 1^st^ attempt	Total correct inhibition trials (incl. after initial failure)	B-trials to criterion	A-trial correct1 = yes 0 = no	Days	Total correct trials	Sessions per day in pre-reversal	TI	Days (2^nd^ value = 4 delay conditions)	Total correct trials			Days	Correct trials
															a	b	a & b combined 4 delay conditions		
M1	Juri	*Callithrix j*.	m	11.2	6	0.10	1.00	5	0	-	-	*0*.*5*	0.22	21/13	0.60		0.58		
M2	Venezia	*Callithrix j*.	f	6.6	5	0.50	0.90	12	0	3	0.67	*0*.*9*	0.00	12/7	0.61		0.62		
M3	Venus	*Callithrix j*.	f	8.0	5	0.45	0.95	5	1	3	0.70	1	0.77	21/13	0.53		0.44		
M4	Verona	*Callithrix j*.	f	7.1	5	0.25	0.90	11	1	3	0.67	1	0.44	10/6	0.57		0.53		
M5	Vesta	*Callithrix j*.	f	7.9	5	0.25	1.00	7	1	3	0.80	1	0.22	8/7	0.57		0.53		
M6	Vito	*Callithrix j*.	m	6.8	6	0.05	0.05	5	0	6	0.73	*0*.*6*	0.33	25/18	0.66		0.56		
M7	Vreni	*Callithrix j*.	f	10.4	5	0.35	0.70	5	1	3	0.77	1	0.00						
M8	Jugo	*Callithrix j*.	m	5.9	7	0.05	0.70	10	0	3	0.77	*0*.*4*	0.10	16/8	0.42		0.43		
M9	Tabor	*Callithrix j*.	m	4.7	6	0.55	0.95	5	1	2	0.70	1	0.00	14/10	0.53		0.55		
M10	Tale	*Callithrix j*.	f	3.8	10	0.30	0.80	5	1	3	0.83	1	0.20	8/6	0.60		0.55		
M11	Tessy	*Callithrix j*.	f	11.6	5	0.00	0.00	7	1	3	0.50	*0*.*9*	0.49	12/6	0.50		0.45		
M12	Thilo	*Callithrix j*.	m	5.0	11	0.50	0.85	5	1	4	0.67	1	0.22	12/6	0.68		0.70		
M13	Kaliper	*Callithrix j*.	m	11.4	6	0.25	0.95	47	0	-	-	*-*	*-*	16/14	0.58		0.56		
M14	Kapi	*Callithrix j*.	m	9.0	6	0.45	1.00	15	0	4	0.70	*-*	*-*	-/10	-		0.51		
M15	Kantor	*Callithrix j*.	m	10.7	6	0.20	0.50	7	1	-	-	*-*	*-*	-/-	-		-		
M16	Kitty	*Callithrix j*.	f	4.6										-/-	-		-		
M17	Lex	*Callithrix j*.	m	8.8										4		0.69	0.69	8	0.13
M18	Nando	*Callithrix j*.	m	2.0										11		0.60	0.60	5	0.04
M19	Nautilus	*Callithrix j*.	m	2.5										9		0.48	0.48	12	0.10
M20	Nebula	*Callithrix j*.	f	2.5										12		0.54	0.54	9	0.21
M21	Nina	*Callithrix j*.	f	8.8										4		0.53	0.53	4	0.46
M22	Nuno	*Callithrix j*.	m	2.0										-		-	-	-	-
M23	Lancia	*Callithrix j*.	f	12.8										4		0.69	0.69	4	0.21
M24	Lexus	*Callithrix j*.	m	12.8										8		0.56	0.56	-	-
M25	Lili	*Callithrix j*.	f	2.0										4		0.58	0.58	4	0.21
M26	Lola	*Callithrix j*.	f	2.3										-		-	-	-	-
M27	Lotus	*Callithrix j*.	m	3.0										-		-	-	-	-
S1	Chipo	*Saimiri s*.	m	9.6	5	0.00	0.00	5	1					4		0.90	0.90	4	0.58
S2	Chris	*Saimiri s*.	m	7.6	5	0.00	0.05	5	1					4		0.60	0.60	4	0.10
S3	Darwin	*Saimiri s*.	m	7.4	6	0.00	0.00	5	1					5		0.56	0.56	6	0.17
S4	Dave	*Saimri s*.	m	8.4	6	0.00	0.00	5	1					4		0.56	0.56	4	0.08
S5	George	*Saimiri s*.	m	6.3	5	0.00	0.00	5	1					4		0.50	0.50	4	0.17
S6	Helio	*Saimiri s*.	m	5.3	5	0.00	0.00	-	-					-		-	-	-	-
S7	Hugo	*Saimiri s*.	m	2.1	6	0.65	1.00	5	1					4		0.79	0.79	4	0.40
S8	Iramo	*Saimiri s*.	m	4.2	5	0.60	1.00	10	1					4		0.42	0.42	4	0.23

All monkeys were captive-born and mother-reared and housed in family (marmosets) and bachelor groups (squirrel monkeys) consisting of two to eight individuals in indoor-and outdoor enclosures. The marmosets’ indoor enclosures had both daylight and artificial light and were composed of one to three components (depending on group size) measuring at least 4 m^3^, each of which was equipped with several climbing structures such as natural branches, a sleeping box, an infrared lamp and a bark mulch floor. Whenever the weather conditions allowed it, each group had free access to an outdoor enclosure. The squirrel monkeys’ indoor enclosures measured 16.55 m^3^ (smaller group) and 24.77 m^3^ (larger group) and were equipped with climbing structures, an infrared lamp and a bark mulch floor. Each group had constant free access to a fully roofed outdoor enclosure, and in addition, the two groups took turns in accessing a larger outdoor area of 86.4 m^3^. The marmosets were fed a vitamin and calcium-enriched porridge in the morning, fresh fruit and vegetables at lunchtime, and gum and mealworms in the late afternoon. In addition, they received a daily protein snack in the afternoon such as pieces of cooked egg. The squirrel monkeys were fed a mixture of pellets and cottage cheese in the morning, a variety of vegetables and a small amount of fruit at lunchtime, and a protein snack such as cockroaches in the late afternoon. Water was available ad libitum from water dispensers (marmosets) or water bowls (squirrel monkeys). All subjects were tested between their regular feedings and never food deprived during the study. Testing time was restricted to 8.30–12.00 hours in the morning and 13.00–16.30 in the afternoon. Each subject was only tested once per day and never during the monkeys’ general resting time from 12.00–13.00 hours. The monkeys could freely enter and leave the test enclosure and were not handled by the experimenter at any time before, during or after the cognitive test sessions. After the completion of this study, the monkeys continued living at the Primate Station, eventually participating in other non-invasive studies.

### General set-up

All subjects were tested individually in a dedicated test enclosure that was connected to their home enclosures through a semi-transparent plastic tube. Once the doors on each end of the tube were opened, the subject could voluntary and freely enter the tube and walk to the test enclosure without being handled by humans at any time. The actual test compartment for the marmosets consisted of a white rear wall, a white floor, two lateral grid walls and a clear Perspex window front. Depending on the cognitive task and respective apparatus, the window front contained one or two rectangular openings through which the subject could reach with its arms to access the apparatus. Each test apparatus was placed on a wooden board (varying size and features) that was mounted on a height-adjustable test table and flush with the test compartment’s window. The test compartment used for the squirrel monkeys also had a white plastic wall in the back and a white floor, and three grid walls. The measurements of the test apparatuses (see S1 Table) were adapted to the squirrel monkeys’ larger body size [28].

### General procedure

The general testing procedure was identical for both species. Before and after each test trial, the experimenter positioned an occluder (a wooden or cardboard sheet) between the test compartment’s window and the test apparatus. Its removal announced the next trial to the subject and the experimenter called the subject’s name and said ‘look’ and made sure that the subject was attentive to the test apparatus and procedure. In the first task (Detour-Reaching), the test apparatus remained within the subject’s reach and was accessible to the subject as soon as the experimenter had removed the occluder between the test compartment’s front and the test apparatus. In all other tasks, the apparatus was mounted on a sliding platform and remained out of the subject’s reach until the baiting process was completed (tasks 2–6) or the time delay had expired (tasks 5–6). The experimenter then pushed the test apparatus into the subject’s reach so that the subject could make its choice. After a correct choice, the experimenter waited for the subject to consume the reward, retracted the test apparatus, set-up the occluder, and immediately continued with the next trial. After a wrong choice, however, the experimenter quickly retracted the test apparatus out of the subject’s reach so that no second choice was possible. She then retrieved the reward, showed it to the subject and placed it in a holding area for re-use in the next test trial. In tasks 1 and 2, the next trial was started immediately after the subject had completed a given test trial. In task 1, this ensured the monkeys’ wellbeing by keeping the duration of each test session as short as possible because a single test trial took up to 2 minutes. In task 2, it was important to quickly continue with the next trial in order to get the subjects into the intended routine of repeatedly choosing the same cup. In tasks 3, 4, 5 and 6, the experimenter only continued with the next test trial once a 15-second (tests 3–4) or 10-second (test 5–6) time interval had expired. This brief delay was intended to make a wrong choice more costly and encourage subjects to concentrate and attempt to choose correctly. Again, since test sessions in tasks 5 and 6 took inherently longer than in the other tasks because each test trial already involved a time delay of up to 30 seconds, the slightly shorter additional inter-trial interval after a wrong choice kept total testing time to a minimum. It is unlikely that this slight difference in inter-trial intervals was perceived by or affected the subjects’ motivation differently because in each task a beep tone announced the end of the delay after a wrong choice. Since food preferences differed between the two species, marmosets received mealworms, crickets or locusts as rewards, whereas squirrel monkeys received mealworms or small pieces of cashew nut.

If a subject lost motivation during a test session and expressed this by refusing to make a choice in 3 consecutive test trials or showed behavioural signs of emotional arousal or avoidance (as determined by the stop criteria in [10]), the experimenter stopped testing and continued the test session on the following day. Importantly, we gave each subject as much time as it needed to complete each task during a total testing period of one to several months.

The criteria to determine the expected amount of testing time in which each task would have had to be completed under an opportunistic testing regime were adopted from a similar study [27] with the exception of tasks 4 and 5a for which these stop criteria would have been too strict. For tasks 4 and 5a, we defined expected testing time as the amount of time in which the majority of subjects had completed the task, because applying the above-mentioned stop criteria would have resulted in most or all subjects being excluded from further testing.

For those subjects who needed longer than expected to complete a task but were not excluded under our full testing regime, we determined the maximum total testing period for each task based on what appeared reasonable depending on the total number of test trials (one to five months). This extended time period allowed subjects ample opportunity to complete each task at its own pace including breaks from testing as required (several days or weeks). We discontinued testing after this time period had expired because it was not justified to continue testing for a completely unlimited time period (i.e. after complete motivation loss).

**Fig 1:**
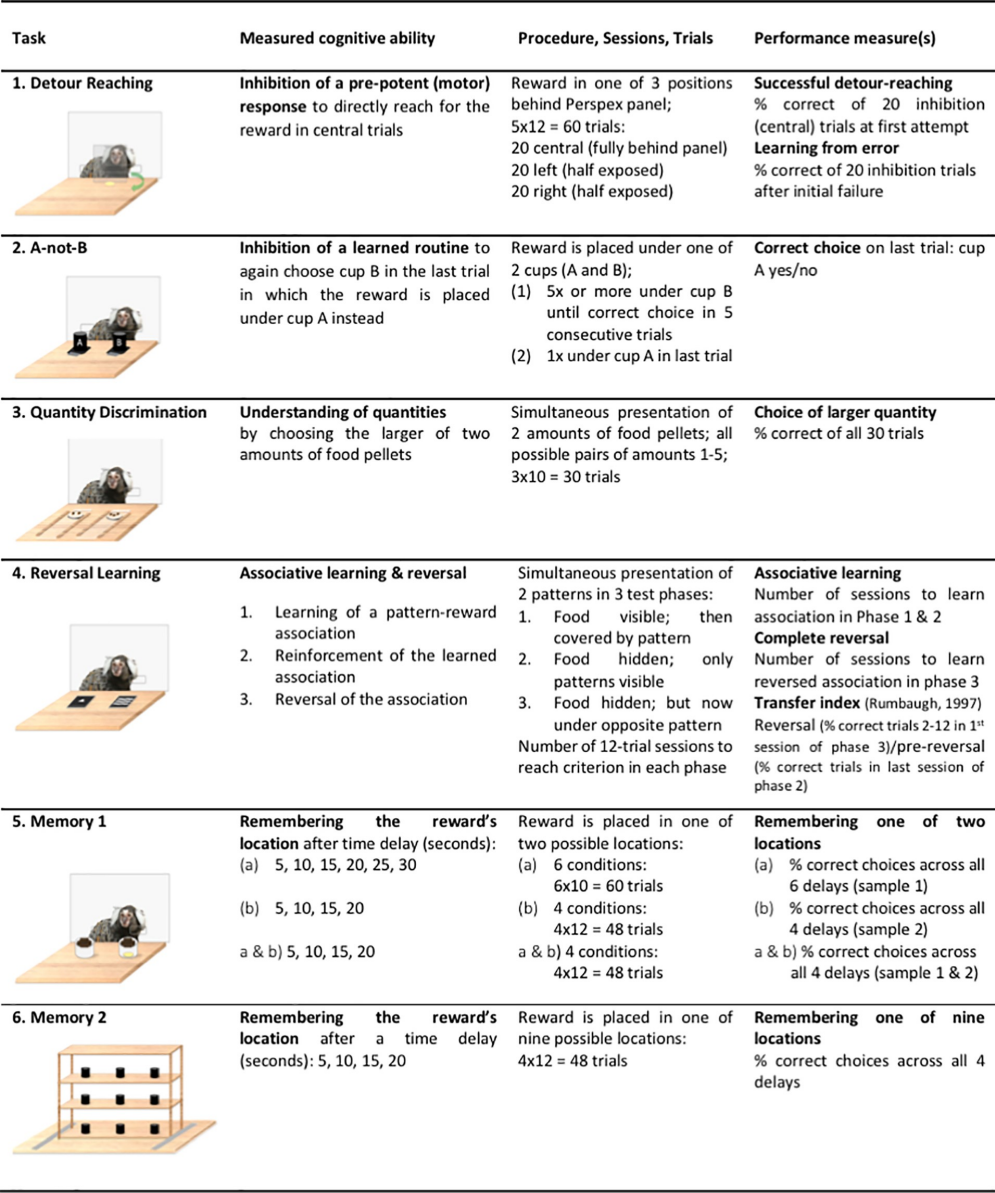
Overview of the 6 tasks of the cognitive test battery.

In this section, and in Fig 1, we provide an overview of the 6 subtests of the test battery (see also Table 2). Further details regarding the test apparatuses are available in S1 Table.

### The cognitive test battery

#### Task 1: Inhibition a–Detour reaching

Measured cognitive ability. Inhibition of a predominant motor response (directly reaching for a food item that is placed behind a transparent barrier) in favour of applying an adequate and successful response (reaching around the barrier to be able to successfully access the food item).

Test apparatus. A static transparent barrier (a quadratic Perspex panel) was vertically attached to the top of a wooden board. The task required the subject to reach around the barrier in order to grasp a food item that was placed behind it.

Familiarisation phase. The subject was given the opportunity to explore the test apparatus in the test compartment without a food reward present for a duration of 10 minutes.

Test phase. In each trial, the experimenter placed the food item on the board and behind the Perspex barrier in one of 3 positions: left, central or right. In the two lateral conditions that simply served as distractors, only half of the reward was positioned behind the left or right-side edge of the Perspex barrier while its other half remained exposed. To successfully retrieve the reward, the subject could directly reach for it and grasp it. In the actual test condition (central trials), however, the reward was placed in the middle of the Perspex barrier so that it was fully occluded by it. To successfully retrieve the reward, the subject had to inhibit directly reaching for the reward as this would have resulted in its hand colliding with the Perspex barrier. It had to reach around the barrier instead so that it could grasp the reward without reaching into the barrier (successful detour-reaching).

If the subject successfully reached around the barrier at its first attempt, the trial was noted as correct. If it failed to do so and reached into the barrier instead, the trial was noted as incorrect. Yet, contrarily to an earlier study [27], the apparatus was kept in place after such initial failure for a maximum of 2 minutes, which allowed the subject the opportunity to make further attempts to reach around the barrier. If a subject eventually succeeded within the 2-minute period, the trial was noted as correct after initial failure and the next trial was started. If the subject did not make any further detour-reaching attempts or still failed to successfully reach around the barrier, the next trial was started after the 2 minutes had expired.

The Detour-Reaching task entailed 5 sessions of 12 trials. In each test session, the reward appeared 4 times in each of the 3 positions (left, central, right) in a counterbalanced and pseudo-randomised order, with the rule that the reward never appeared in the same location on more than two consecutive trials. This generally resulted in the completion of the full 5 test sessions and 60 trials in 5 consecutive days. However, subjects who did not complete a whole session or refused to participate at all on a given day were tested the following day. This resulted in the test sessions being spread over more than the expected 5 days. We stopped a test session on a given day if a subject was not motivated to complete all trials. However, we allowed these subjects to eventually complete all 5 sessions of the task in as many testing days as it needed.

Outcome variables. (1) Successful detour-reaching: per cent correct trials of the 20 central trials at first attempt. (2) Learning from error: per cent correct trials at first attempt plus after initial failure (further detour-reaching attempts).

Stop criterion in opportunistic testing. In opportunistic testing, the five sessions of the Detour-Reaching task would have been supposed to be completed within five consecutive days (see Occluded Reach task, [27]).

Of the 23 subjects in our study sample who completed the Detour-Reaching task, nine of the 15 marmosets (M1, M6, M8, M9, M10, M12, M13, M14, and M15) and 3 of the 8 squirrel monkeys (S3, S4, and S7) needed longer than five testing days to complete the five test sessions. These 12 subjects would have been excluded from (further) testing owing to motivational issues, had we stopped testing after five consecutive days as in opportunistic testing.

In our full testing, in which we gave the monkeys a total testing time period of 1 month to complete the Detour-Reaching task, all 12 individuals who needed more testing time than expected eventually also completed the task and no subject had to be excluded.

#### Task 2: Inhibition b–A-not-B

Measured cognitive ability. Inhibition of a learned routine, i.e. to avoid choosing the same location that had consistently been rewarded and switching to a second location instead that was now being rewarded. In order to successfully choose the correct cup, the subject had to inhibit a learned association between cup B and the food reward.

Test apparatus. Two opaque black plastic cups (A and B) were placed upside-down on a wooden sliding platform. The cups were attached to the board in a way that the experimenter could easily open and close them by flipping them back and forwards.

Familiarisation. The subjects learned (1) how to open the cups, (2) that only one cup at the time would contain a reward, and (3) that only one choice was possible in each trial.

Test. The test consisted of 6 trials conducted in a single session. During the first 5 trials, the experimenter placed a reward under cup B while the subject was watching, closed both cups simultaneously and quickly pushed the test apparatus within the subject’s reach. The subject could then reach out to choose one of the two cups, by lifting it or attempting to do so. In the 6^th^ trial, the experimenter placed the cricket under cup A instead of B.

Cup A was only baited once a subject had correctly chosen cup B in 5 consecutive trials. If the subject did not correctly choose cup B in the first 5 consecutive trials, the experimenter repeated baiting cup B until the subject succeeded. If a subject did not make 5 consecutive correct choices within a single test session of 14 trials, it was tested again the following day. If a subject stopped participating during the session, it was also tested the following day.

Outcome variable. Correct choice in the last trial (cup A instead of B): yes/no.

Stop criterion in opportunistic testing. In opportunistic testing, testing with subjects who failed to choose cup B in the first 5 consecutive trials would have been stopped and those subjects would have been excluded from further analysis.

In our study, this would have resulted in losing 9 of the 22 subjects in the analysis who participated in the actual test. Eight of the 15 marmosets (M2, M4, M5, M8, M11, M13, M14 and M15) and one of the 7 squirrel monkeys (S8) did not achieve 5 correct B-choices in the initial 5 consecutive B-trials and therefore needed longer to succeed in 5 consecutive B-trials.

In our full testing approach in which we gave the monkeys one month (including the familiarisation phase) to complete the A-not-B task and as many B trials as needed, all 9 subjects who needed more than 5 B-trials to reach criterion eventually did so and completed the actual test. We only had to exclude one other squirrel monkey (S7) because he still refused to complete the familiarisation trials once all other subjects had completed the full task.

#### Task 3: Quantity discrimination

Measured cognitive ability. Understanding of quantities by distinguishing between pairs of different quantities, i.e. two amounts of edible items.

Test apparatus. Schmitt & Fischer [22] had reported that two monkey species performed best when the two quantities to choose from consisted of (1) inedible items (tokens), or (2) edible items that differed from the reward items in food type. In our study, we therefore used monkey chow pellets as stimulus items and crickets as a reward for a correct choice. Pellets are interesting enough to attract the marmosets’ attention because they are edible but not desirable enough to distract them from the task at hand. However, in contrast to other studies [22, 27], the subjects in our study were not allowed to consume as many items as they had chosen, regardless of whether their choice was correct. Rather, the marmosets were rewarded with a single cricket if they correctly chose the larger pellet quantity in a given test trial and received no reward for an incorrect choice.

Pre-test. The subjects were presented with a single and obvious numerical contrast (1 vs. 6 pellets), and the location of the larger quantity was counterbalanced and pseudo-randomised for all contrasts. This ensured all subjects understood they had to choose the larger of two pellet quantities in order to obtain the cricket. Before proceeding to the test phase, they had to reach the criterion of ≥ 80% correct trials within a single pre-test session of 10 trials.

Test. The test consisted of 3 sessions of 10 trials each. In each session, the subjects were presented with 10 different numerical contrasts (all possible quantity combinations of the 1–5 pellets). As in the pre-test phase, the location of the larger quantity was counterbalanced and pseudo-randomised for all contrasts.

Outcome variable. Per cent correct trials (choice of the larger amount) across all 3 test sessions.

Stop criterion in opportunistic testing. Under an opportunistic testing regime, testing would have been stopped if a subject did not complete the 30 trials within 3 sessions that were conducted on 3 consecutive days and this subject would have been excluded from the analysis.

Of the 15 marmosets who had entered the pre-test, 12 subjects participated in the actual Quantity Discrimination test. Nine subjects (7 females and 2 males) completed all 3 test sessions within the expected time frame of 3 testing days (one male subject even within 2 days). The remaining 3 male subjects needed up to twice as long to complete the task (M12 and M14 took 4 testing days, M6 took 6 days) and would have been excluded in opportunistic testing.

Under our full testing regime, in which we gave the monkeys one month (including the pre-test) to complete the task, the 3 above mentioned male individuals also completed the task. The only individuals we had to exclude were 3 other males (M1, M13, and M15) who were still not motivated to fully participate in the pre-test after a total testing period of one month and once their conspecifics had completed the whole task.

#### Task 4: Reversal learning

Measured cognitive ability. Cognitive flexibility, i.e. reversing a previously learned association to a new one if the previously learned association is no longer rewarded.

Test apparatus. The apparatus consisted of a wooden board that contained two food wells and two rectangular wooden plates with two distinct black and white patterns printed on top.

Familiarisation phase. Each subject was familiarized with the basic setup and procedure in order to ensure it understood that it needed to uncover the baited well to obtain the reward by pushing away the correct plate. In this phase, both wooden plates looked identical (no patterns). In each trial, the experimenter placed a cricket in full sight of the subject in one of the two food wells and covered both wells with the plates. Pushing away the plate from the baited well allowed the subject to retrieve the cricket. To pass to the test phase, the subjects had to reach the criterion of 10/12 correct trials.

Test Phase 1 –Unhidden baiting–Learning of an association. The subjects were allowed to watch the baiting process to learn the association between one of the two patterns and the food reward. As in the familiarisation phase, the now patterned plates were initially each positioned behind one of the two food wells. The experimenter then placed a cricket in one food well and simultaneously covered both wells with the patterned plates. The reward was always under the same pattern and only choosing the correct one of the two patterns was rewarded. The criterion to move on to the next phase was again 10/12 correct trials.

Test Phase 2 –Strengthening of the learned association. The experimenter baited the food well with the same pattern as in phase 1 but now behind the occluder so that the subject could not observe the baiting process. As in phase 1, she simultaneously placed both patterned plates on top of the two food wells so that the subject could not make use of any auditory cues. She then removed the occluder and the subject could make its choice. The criterion to move on to the next phase was again 10/12 correct trials.

Test Phase 3 –Reversal of the learned association. The experimenter followed the same procedure as in phase 2 with the crucial difference that she now always placed the food reward under the opposite pattern (i.e. the one that had previously not been rewarded). The criterion was again 10/12 correct trials.

Outcome variables. Number of sessions to reach criterion (80% correct trials within a single session) in each of the 3 test phases: (1) learning the association between one pattern and the food reward, (2) strengthening of the learned association and (3) reversal of the learned association. In addition, we used a cognitive flexibility measure, the *Transfer Index (TI)* [29, 30], which puts a subject’s performance in the first session of phase 3 (reversal) in relation to its performance in the last session of phase 2.

Stop criterion in opportunistic testing. Had we used an opportunistic testing regime, subjects who did not complete each test phase in a maximum of 7 sessions would have been excluded from further testing. Since none of the marmosets in our study completed phase 3 within 7 sessions, this approach would have resulted in eventually losing the whole study sample.

In our full testing approach, we did not use such a strict criterion but we discontinued testing with subjects who (1) needed more than 19 test sessions of 12 trials in the last of the 3 test phases (reversal) or (2) failed to complete phase 2 or 3 owing to motivational issues over a total testing period of 5 months. Of the 15 marmosets who participated in the Reversal Learning task, only one male subject (M13) lost motivation very early on and failed to complete phase 1. Of the 14 remaining subjects, two males (M14 and M15), did not complete phase 2, despite having reached criterion very quickly in phase 1. Finally, of the 12 subjects who had completed phase 2 and entered phase 3, five subjects could not be tested until completion of the reversal: three males (M1, M6 and M8) who completely refused to participate owing to motivational issues and two females (M2 and M11) who developed a strong side bias in phase 3 which they did not overcome. However, since all 12 subjects had completed the first session of phase 3, the *TI* could be determined.

#### Task 5: Memory 1 –traditional task format with two choice options

Measured cognitive ability. Remembering in which of two locations a food reward has been hidden over increasing time delay periods.

Memory task 1a). In the traditional two-choice Memory task version 1a, the test apparatus and procedure largely corresponded to the Hidden Reward Retrieval test in [27]. Two white plastic containers were attached to a wooden board that could be slid into the subject’s reach. Both containers were filled with dark bark mulch and a small piece of a yellow locust was used as a reward in each trial.

Memory task 1b). In the two-choice Memory task version 1b, the test apparatus consisted of two black containers with loosely placed lids that were attached to the middle one of three wooden platforms held together by a wooden frame (for details see [24]).

Pre-test. A no-delay condition was used as a pre-test. In this pre-test, the subjects could witness the baiting and choose immediately afterwards, without delay. Pre-test criterion was reaching ≥ 80% correct choices in a single 10-trial session. This ensured the subjects understood the task at hand before being exposed to time delay.

Test. At the beginning of each trial, both containers were covered with a rectangular piece of bark mull in Memory task 1a or the containers were closed in Memory task 1b. Once the subject was attentive, the experimenter did the following: In Memory task 1a, she removed the cover of one of the containers, placed a piece of a locust in the container and put the cover back in place so that the bright yellow locust was no longer visible and both containers looked the same. In Memory task 1b, the experimenter lifted the lid of one of the two cups, placed a mealworm in the cup and closed it so that the mealworm was no longer visible to the subject. During the time delay period (5–30 seconds in Memory task 1a and 5–20 seconds in Memory task 1b), the test apparatus remained out of the subject’s reach. As soon as the time delay had expired, the experimenter pushed the test apparatus into the subject’s reach allowing it to choose one of the two containers. The subjects were tested with one session of 10 (Memory 1a) or 12 (Memory 1b) trials per day. A correct choice consisted of uncovering (Memory 1a) or opening (Memory 1b) the baited container.

Outcome variable: Number of correct trials (per cent correct choices) across all delay conditions in Memory 1a (6 delays, 60 trials) and Memory 1b (4 delays, 48 trials), as well as in both task versions combined (4 delays, 40 or 48 trials, all subjects from a and b).

Stop criterion in opportunistic testing.

Memory 1a). In opportunistic testing, subjects would have been excluded if they did not complete all 6 test sessions in the expected 6 testing days (Hidden Reward Retrieval, [27]). Since none of the subjects in marmoset sample 1 who participated in this two-choice memory task fulfilled this criterion, they would all have been excluded from further testing and no statistical analysis would have been possible.

However, the majority of subjects completed the task within 12 testing days which we set as expected testing time instead.

In our full testing approach, we gave each marmoset as many test sessions as it needed to complete the pre-test and the 6 test sessions during a 5-month testing period. Of the subjects who needed more than 12 testing days, five (M1, M3, M6, M8, and M13) eventually completed Memory task 1a. However, three other marmosets (M14, M15, and M16) had not completed the whole task once the 5-month testing period expired and had to be excluded from statistical analysis.

Memory 1 b). In opportunistic testing, subjects would have been excluded if they failed to complete the 4 test sessions in the expected 4 testing days. This would have resulted in excluding 4 of the 8 marmosets (M18, M19, M20, and M24) and one of the 8 squirrel monkeys (S3) who participated in Memory task 1b.

We gave each subject a 5-month testing period (including the pre-test) to complete the memory task. Fifteen of the 19 monkeys (8 of the 11 marmosets of study sample 2 and 7 of the 8 squirrel monkeys) who had entered Memory task 1b eventually completed the full task within the given testing period. The remaining 3 marmosets (M22, M26, and M27) and one squirrel monkey (S6) had lost motivation early in the pre-test or after a very small number of test trials and the experimenter could not regain their motivation to fully participate. Using our full testing approach, we thus only had to exclude these 4 subjects from statistical analysis who were not sufficiently motivated to participate in the actual test.

Since the two versions of Memory test 1 slightly differed in terms of used methodology which could potentially have affected the results, we first analysed and reported them separately. In a third analysis, we then also combined the subjects of marmoset sample 1 who had completed the first 4 test sessions of Memory task 1a and the subjects of marmoset sample 2 and the squirrel monkeys who had completed Memory task 1b.

Using our full testing approach in which we had given each subject sufficient time to complete the (first) four delay conditions of Memory task 1, we obtained performance scores for one more male marmoset from sample 1 (M14) who had dropped out in Memory task 1a because he had not completed all 6 delay conditions. Therefore, we only had to exclude two subjects of study sample 1 (M15 and M16) who had not completed all trials of the 4 test sessions after expiration of the 5 months testing period.

Three other marmosets (M22, M26, and M27) of study sample 2 and one squirrel monkey (S6) also had to be excluded because they had dropped out in the pre-test phase or after only a few test trials owing to motivational issues.

#### Task 6: Memory test 2 –Optimised test format with nine choice options

Measured cognitive ability. Remembering in which of nine locations a food reward has been hidden over increasing time delay periods.

Apparatus and procedure. This memory task differed from task 5 in that nine rather than two options were available to choose from. The apparatus and procedure were identical to the ones used in Memory task 1b with the only difference being that 9 rather than only 2 cups were used. The method has been described in detail in an earlier publication [24].

Outcome variable. Per cent correct choices across all delay conditions (4 delays, 48 trials).

Stop criterion in opportunistic testing. In opportunistic testing, subjects would have been excluded if they had not completed the 4 delay conditions in 4 sessions conducted on 4 consecutive days.

In our full testing approach, we continued testing for a total testing period of 5 months in order to allow every subject to complete the test. Seven of initially 11 marmosets and 7 of initially 8 squirrel monkeys completed the whole Memory task 2 whereas one marmoset (M24) only completed the first test session (the 5-second delay condition). Three marmosets (M22, M26, and M27) and one squirrel monkey (S6) eventually had to be excluded because they had either not completed the pre-test or only a very small number of test trials before they refused to participate at all.

## Data analysis

For each of the six cognitive tasks, we analysed whether individuals who finished the task in the expected time (i.e. would have been included in opportunistic testing) differed in performance from individuals who needed longer (i.e. would have been excluded in opportunistic testing but were included in our full testing). We ran Generalised Linear Mixed Models (GLMMs), with the exception of the A-not-B task (task 2) for which we computed Fisher exact tests owing to the small number of trials in this task. The outcome variables for cognitive performance were the response variables, and subject was included as a random factor in all models. Testing time needed to complete a given test was included as a binary fixed factor: expected amount of time vs. longer. Individual test session, species (where applicable) and all two-way interactions were also included as fixed factors in the models. We calculated all models with biologically meaningful factor combinations and identified the best model using the Akaike criterion corrected for small sample sizes (AICc, [31]).

## Results

### 1. Detour reaching

Of the 23 individuals who completed the Detour-Reaching task, 11 individuals completed the task in time (≤ 5 days; 6 marmosets, 5 squirrel monkeys) whereas 12 individuals took longer than expected (> 5 days; 9 marmosets, 3 squirrel monkeys). The best model to explain performance in the Detour-Reaching task included the fixed factors test session (*F*(4, 84) = 8.48, *p* = .000), species (*F*(1, 21) = 1.26; *p* = .274), and their interaction (session*species: *F*(4, 84) = 5.51, *p* = .001). Thus, individuals who finished the task in the expected time did not differ in performance from individuals who took longer to complete it (see Fig 2.1). The interaction session*species indicates that while the marmosets improved their performance over the course of the five sessions and therefore learned to inhibit reaching directly for the food, this was not the case for the squirrel monkeys (see S1 Fig).

**Fig 2:**
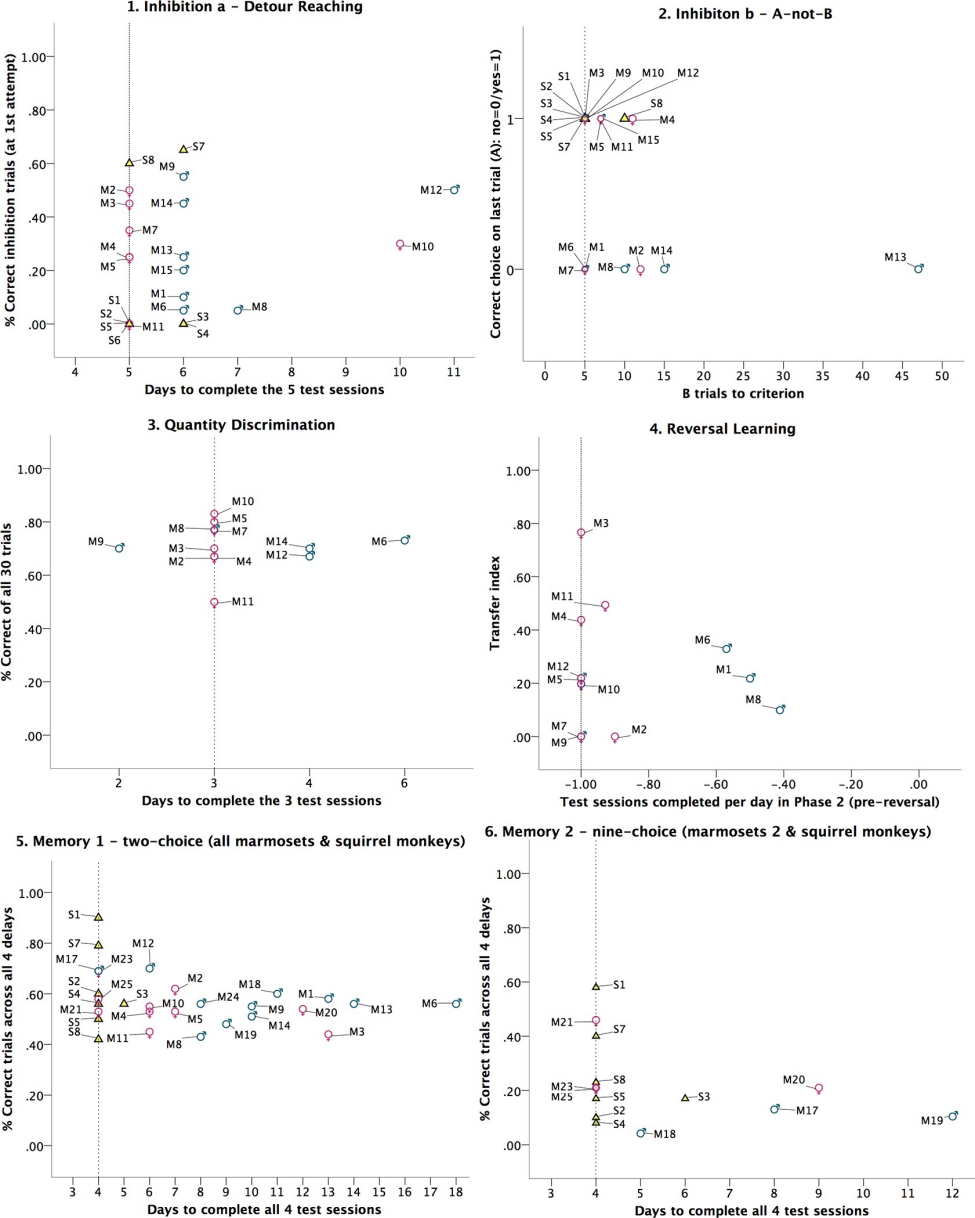
The subjects’ performance in the 6 cognitive tasks depending on how much testing time they needed to complete each task. The dotted line marks the expected amount of time after which testing would have been discontinued and subjects who needed longer would have been excluded had we used the classical opportunistic approach. Marmosets are represented by ID numbers M1-M27 and gender symbols, squirrel monkeys by ID numbers S1-S8 and yellow triangles.

### 2. A-not-B

Of the 22 individuals who completed the A-not-B task, 13 individuals (7 marmosets, 6 squirrel monkeys) completed the test trials in time (five B-trials to criterion). Nine individuals (8 marmosets, 1 squirrel monkey) took longer than the expected amount of testing time (> five B-trials) to reach criterion (5 consecutive correct B-trials) before receiving the final (A) trial. Overall, 8 of the 15 marmosets and all 7 squirrel monkeys who completed the A-not-B task correctly chose cup A in the last trial and therefore passed the task whereas the remaining 8 marmosets failed it. Since a GLMM was not applicable owing to the relatively small number of test trials, two Fisher exact tests were conducted instead. The first Fisher exact test determined whether testing time (the number of B trials required to correctly choose cup B in five consecutive trials) affected the subjects’ performance (whether they correctly chose cup A in the last trial). The second test determined whether the species difference in performance was significant. Although there was a non-significant trend that subjects who needed more time to complete the A-not-B task were more likely to fail it, testing time had no significant effect on the monkeys’ performance (Fisher’s exact test; *p* = 0.178). Likewise, there was a non-significant trend for squirrel monkeys to outperform marmosets (Fisher’s exact test; *p* = 0.121) (see Fig 2.2).

### 3. Quantity discrimination

Of the 12 marmosets who completed the Quantity Discrimination task, nine individuals did so in time (≤ 3 days; 7 females, 2 males) whereas 3 individuals took slightly longer than expected (> 3 days; 3 males). Overall, the 12 subjects who completed the Quantity Discrimination test performed well above chance (*Mean* score = 71% correct choices, *SD* = 8%; *t*(11) = 8.53, *p* = .000). The best model to predict cognitive performance was the one that only included one fixed effect, testing time, which did not affect the marmosets’ performance (*F*(1, 10) = 0.42; *p* = 0.534) (see Fig 2.3).

### 4. Reversal learning

For the Reversal Learning task, we first report how long (number of test sessions) the subjects needed to reach the learning criterion (≥ 80% correct trials within a single session) in each of the 3 test phases. We also report whether the number of days the subjects needed on average to complete a 12-trial test session affected their performance (per cent correct trials in the last session). Finally, we answer the central question: whether the amount of testing time (in this task measured as the number of completed trials per testing day) affected the subjects’ cognitive flexibility (as measured by the *TI* [29]).

#### Phase 1—Learning of an association

Of the 14 marmosets who completed phase 1, all individuals did so in time whereas no subject took longer than expected. In phase 1, the 14 marmosets reached criterion (≥ 80% correct trials in a single session) within an average of 2 test sessions (*M* = 2.07, *SD* = 1.60), with the minimum being 1 session and the maximum 6 sessions (see S2 Fig).

#### Phase 2—Strengthening of the learned association

Of the 12 marmosets (7 females and 5 males) who completed phase 2 (strengthen the learned association), seven (4 females and 3 males) did so in time whereas 5 (3 females and 2 males) needed longer than expected. The number of 12-trial sessions needed to complete phase 2 ranged from 4 to 13 sessions, with the average being 7 sessions (*M* = 6.75, *SD* = 3.11) (see S2 Fig).

#### Phase 3 Reversal of the learned association

Of the 7 marmosets (4 females and 3 males) who completed phase 3 (full reversal of the initially learned association), only 1 female subject (Vesta) did so in time whereas 6 subjects (4 females and 2 males) needed longer. The marmosets completed phase 3 in 7 to 19 sessions, with the average being 13 sessions (*M* = 12.57, *SD* = 4.96) (see S2 Fig).

#### Transfer Index *(TI)*

All of the 12 marmosets who had entered phase 3, completed at least the first test session of the reversal. To compare cognitive flexibility between individuals, the *TI* was calculated for each of these subjects. In order to determine whether testing time, i.e. the number of days a subject needed to complete a session in phase 2 (expected = 1 session of 12 trials per day; longer = < 1 session of 12 trials per day) or a subject’s sex had an effect on its cognitive flexibility (as measured by the *TI*), we ran GLMMs with the fixed effects time, sex, and the interaction of these two factors. The two models that only included testing time and sex, respectively, only differed marginally from each other (ΔAIC*c* < 1). Neither time (*F*(1, 10) = 0.62, *p* = 0.808) nor sex (*F*(1, 10) = 0.91, *p* = .362) significantly affected a subject’s *TI* (see Fig 2.4).

### 5) Memory 1 –Traditional two choice task

#### Version a) Marmoset sample 1

Of the 12 marmosets (6 females and 6 males) who completed the 2-choice Memory task 1 a (study sample 1), 6 subjects did so in our set time (≤ 12 days) whereas 6 subjects took longer (> 12 days). The best model to predict whether time needed to complete the task had an effect on the monkeys’ performance only included the fixed effect time which did not have a significant effect on performance (per cent correct trials): *F*(1, 10) = 0.71; *p* = 0.420. The two models that only differed marginally from the best model (ΔAICc < 1) each also included only one fixed effect, delay and sex respectively. Delay condition had a significant effect on a subject’s performance (*F*(1, 5) = 3.44; *p* = .009) whereas a subject’s sex did not (*F*(1, 10) = 0.17; *p* = 0.690) (see S3 Fig).

#### Version b) Marmoset sample 2 & squirrel monkeys

Of the 15 monkeys (8 marmosets and 7 squirrel monkeys) in study sample 2 who completed the two-choice memory task, eleven individuals (5 marmosets, 6 squirrel monkeys) did so in time (≤ 4 days) whereas the other 5 individuals (4 marmosets, 1 squirrel monkey) needed longer (> 4 days).

The best model only included the fixed factor time which did not have a significant effect on the monkeys’ performance (*F*(1, 18.66) = 0.38; *p* = .544) (see S3 Fig).

#### Extended study sample (all subjects from both task versions a and b)

When combining the (first) four delay conditions of those marmosets (sample 1 and 2) and squirrel monkeys who had completed all essential trials of the two-choice memory task, we obtained performance scores for two more male marmosets from sample 1 (Kapi and Kantor) who had dropped out in Memory task 1a because they had not completed all 6 delay conditions. Of the 28 monkeys (7 squirrel monkeys and 21 marmosets) who completed the four delay conditions, ten (4 marmosets and 6 squirrel monkeys) did so within the expected time (4 days) whereas 18 (1 squirrel monkey, 17 marmosets) took longer (> 4 days). The best model only included the fixed factor delay which had a highly significant effect on the monkey’s performance (*F*(3, 81) = 5.33, *p* = .002). Although the next best model also included time, required testing time did (as in all other models) not significantly affect performance (*F*(1, 26) = 3.00, *p* = 0.095) (see Fig 2.5).

#### 6. Memory 2 –Optimised nine-choice memory task (marmosets 2 & squirrel monkeys)

Of the 14 monkeys (7 marmosets of study sample 2 and 7 squirrel monkeys) who completed the nine-choice memory task, nine individuals (3 marmosets, 6 squirrel monkeys) did so in time (4 days) whereas the other 5 individuals (4 marmosets, 1 squirrel monkey) needed longer (> 4 days). Time to complete the 9-choice memory task (4 days versus > 4 days) did not affect the subjects’ performance in the 9-choice memory task (no significant effect in any of the models). The best model only included the fixed effect delay which did affect performance (i.e. the proportion of correct choices): *F*(1, 3) = 5.48; *p* = .003, indicating that memory performance decreased with increasing retention delay. While species had no effect on overall performance in any of the models, there was a significant interaction of delay and species in the third-best model (see Fig 2.6).

## Discussion

The main question was whether the amount of testing time a subject required to complete a task affected its cognitive performance in that task. Our results show that this was not the case and that in each task, the performance of subjects who needed longer than expected to complete the task did not differ from those who completed the task in time.

It is important to stress that even in our full testing approach, we lost subjects from the sample. This was because the monkeys were never forced to enter the testing enclosures but had to be at least sufficiently motivated to approach the testing area voluntarily to be included. While we had made every effort to allow each individual to complete each task at its own pace, it was not justified to continue testing for a completely unlimited time period. Therefore, we cannot rule out the possibility that the few subjects who dropped out in some tasks despite being given ample time to complete them, differ in cognitive ability from subjects who completed the tasks.

Despite this possible exception, our findings suggest that opportunistic testing in primates (i.e. only testing subjects who readily participate) does not bias the results of cognitive tasks in several physical domains, at least in marmosets and squirrel monkeys. To what extent they generalise to tasks from the social domain or to paradigms that do not require subjects to choose between a set of presented options remains to be established.

Species differences in cognitive performance were minor and mostly non-significant, even though we cannot exclude that this may be owing to the relatively low number of squirrel monkeys in our sample. The only significant effect was that in the detour-reaching task, marmosets increased their performance over time, whereas squirrel monkeys did not, which led to increased inhibition performance in marmosets in later trials (see S1 Fig). One possible explanation is that the marmosets are better at inhibiting a direct reach for food because, as cooperative breeders, they frequently share food with immature group members including food offering and calling others to a food source [32]. Food sharing, and in particular proactive food offering, arguably requires the ability to inhibit the immediate impulse to take and consume the food. To further test this possibility, future tasks could compare whether marmosets who are more inclined to share food indeed show stronger detour reaching performance. Intriguingly, in the second inhibition task, the A-not-B task, marmosets did not outperform the squirrel monkeys but there was rather a trend in the opposite direction. It thus appears that the two inhibition tasks measure different aspects of inhibitory control. This possibility is consistent with MacLean et al [33] who tested a large number of primate (and other) species with the same two inhibition tasks and likewise did not find a particularly strong correlation between the two.

Our finding that the selection bias that results from opportunistic testing does not affect cognitive performance in marmosets and squirrel monkeys in several physical cognition tasks adds to the list of non-cognitive factors that have been studied so far regarding their potential to affect cognitive performance (Table 1). While required testing time did not affect cognitive performance in our study, another testing-related factor, task format, did affect cognitive performance of non-human primates [22–25] and it sometimes did so differently in different species [22, 23]. Although the effects of subject-related factors (such as individual differences in personality traits or housing conditions) seem less ambiguous, the overall pattern remains inconclusive.

To sum up, we showed that at least for two New World monkey species and the given set of cognitive tasks, opportunistic testing does not bias the test results. If our findings generalise to other non-human primate species and cognitive tasks, then maximising sample sizes by testing only consistently motivated subjects will be a valid alternative whenever a sufficiently large number of subjects is available but full testing is not feasible owing to time constraints.

Future studies should extend the list of non-cognitive factors that affect cognitive performance, so that the validity and reliability of existing cognitive tests can be further improved and newly developed tests will be less prone to performance biases. Once this task is achieved, cognitive test batteries can then be used more reliably to compare the cognitive abilities of non-human primate individuals and species.

## Ethics statement

This study was performed in accordance with the Swiss legislation and licensed by the Veterinary Office of the Canton of Zurich (Licence number 183/13, 24826, degree of severity: 0, i.e. no harm). Thus before, during and after this study, the monkeys were never constrained or subjected to any pain, suffering or injury and their general state of health was not impaired. All cognitive tasks were conducted non-invasively between the monkeys’ regular feeding times. The monkeys could freely enter and leave the test enclosure without being handled by humans at any time and were never isolated from their social groups. After the completion of this study, the monkeys continued living at the Primate Station, eventually participating in other non-invasive studies.

## Supporting information

S1 Table Materials & measurements for the tasks of the cognitive test battery. Each test apparatus was placed on a wooden board (varying size and features) that was mounted on a height-adjustable test table and flush with the test compartment’s window. Depending on the cognitive task, the Perspex window front used for the marmosets contained one or two openings with rounded edges at a distance of 10 cm above the test compartment’s floor: (1) one large opening (18 x 2.5 cm), (2) two small openings: 4 x 2.4 cm, 12 cm apart.
(PDF)

S1 Fig Performance in the 5 test sessions of the Detour-Reaching task. Successful detour-reaching in the inhibition trials at (a) first attempt and (b) after initial failure to reach around the transparent barrier. Marmosets (grey solid bars)/squirrel monkeys (yellow dotted bars).
(PDF)

S2 Fig Number of test sessions to criterion in the 3 phases of the Reversal Learning task. Fewer (12-trial) sessions indicate better performance, hence the negative prefix on the y-axis. Phase 1—learning of the initial association between a pattern and a reward. Phase 2—strengthening of the learned association. Phase 3—reversal of the learned association.
(PDF)

S3 Fig The subjects’ performance in the two versions of Memory task 1 depending on how much testing time they needed to complete the task. The dotted line represents the expected amount of time (number of testing days) after which testing would have been discontinued and subjects who needed longer would have been excluded had we used the classical opportunistic approach. (a) The marmosets of study sample 1 (represented by ID numbers M1-M16 and gender symbols) were tested with version a of Memory 1 (adapted from Banerjee et al., 2009), (b) the marmosets of study sample 2 (ID numbers M17-M27 and gender symbols) and the squirrel monkeys (represented by yellow triangles) were tested with version b (Schubiger et al., 2016). The two versions of Memory 1 slightly differed in details of experimental set-up (apparatus) and design (number of delay conditions; also see Fig 1 and S1 Table).
(PDF)
