# Supplementary material for: Does opportunistic testing bias cognitive performance in primates? Learning from drop-outs
Source: PLoS One. 2019 Mar 20;14(3):e0213727. doi: 10.1371/journal.pone.0213727 (PMC6426242; doi:10.1371/journal.pone.0213727)
Supplement: S1 Table — Each test apparatus was placed on a wooden board (varying size and features) that was mounted on a height-adjustable test table and flush with the test compartment’s window. Depending on the cognitive task, the Perspex window front used for the marmosets contained one or two openings with rounded edges at a distance of 10 cm above the test compartment’s floor: (1) one large opening (18 x 2.5 cm), (2) two small openings: 4 x 2.4 cm, 12 cm apart. (PDF) [file pone.0213727.s001.pdf]

S1 Table. Materials & measurements for the tasks of the cognitive test battery. Each test apparatus was placed on a wooden board (varying size and features) that was mounted on a height-adjustable test table and flush with the test compartment's window. Depending on the cognitive task, the Perspex window front used for the marmosets contained one or two openings with rounded edges at a distance of 10 cm above the test compartment's floor: (1) one large opening (18 x 2.5 cm), (2) two small openings: 4 x 2.4 cm, 12 cm apart.

Task	Item	Measurements (in cm)	
		Marmosets	Squirrel monkeys
1.Detour-Reaching 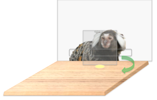	Window front (1)	18.0 x 2.5	
	Wooden board with an indentation (1cm deep) for Perspex panel	34.0 x 37.0	42.0 x 46.0
	Transparent Perspex panel (0.5 cm in thickness); quadratic surface exposed	8.0 x 9.0	13.5 x 14.5
	Reward: mealworm (or cricket)		
2. A-not-B 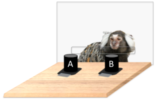	Window front (1)	18.0 x 2.5	
	Wooden board	34.0 x 37.0	42.0 x 46.0
	2 black plastic cups (each with a silver insulating strip on its base)	Ø 2.6, h. 3.8	Ø4.5, h: 5.2
	Reward: mealworm (or cricket)		
3. Quantity Discrimination 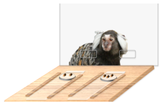	Window front (2)	18.0 x 2.5	
	Wooden board<br>with 4 wooden sliding rails for sliding platforms	41.0 x 40.0 2.0<br>x 33.0	
	2 transparent plastic sliding platforms<br>with white plastic handles on their front ends	28.0 x 7.0<br>1.0 x 7.0	
	2 small petri dishes with white floors (plastic foil)<br>and transparent lids	Ø 5.2, h: 1.2<br>Ø 5.6, h: 0.7	
	2 white flat cotton pads to keep the pellets in place	Ø 5.0	
	Experimental stimuli: monkey chow pellets	Ø 0.5, l: 1.5	
	Rewards: crickets		
4. Reversal Learning 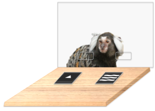	Window front (2)		
	1 wooden sliding board with<br>2 round food wells at 2 cm distance from board's front	33.5 x 32.5<br>Ø 5.1, h: 1.6	
	2 wooden plates, each with pattern (=paper covered with plastic foil):<br>1) white triangle on black background<br>2) alternating black and white "waves"	7.5 x 6.5	
	Rewards: crickets		
5. Memory 1a 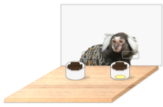	Window front (2)		
	1 wooden sliding board with<br>2 round food wells at 2 cm distance from board's front	33.5 x 32.5<br>Ø 5.1, h: 1.6	
	2 white round plastic containers (11 cm apart)<br>filled with ramial chipped bark mulch	Ø 5.1, h: 3.1	
	2 cover plates (= larger rectangular bark mulch pieces of equal size)		
	Rewards: yellow desert locusts		
5. Memory 1b 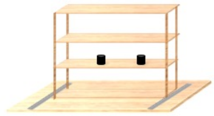	Wooden sliding board	45.0 x 30.0	95.0 x 50.0
	Wooden frame with 3 platforms	w 40 x h 37.5	w 80.0 x h 75.0
	2 black plastic cups with grey lids	Ø 3.1, h: 1.1	Ø 3.1, h: 2.3
	2 Velcro tape strips		
	Rewards: crickets or small pieces of a cooked apple (M); mealworms or small pieces of cashew nut (S)		
6. Memory 2 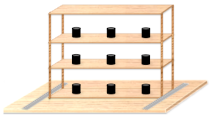	Wooden sliding board	45.0 x 30.0	80.0 x 75.0
	Wooden frame with 3 platforms	w 40 x h 37.5	w 80.0 x h 75.0
	9 black plastic cups with grey lids	Ø 3.1, h: 1.1	Ø 3.1, h: 2.3
	9 Velcro tape strips		
	Rewards: crickets or small pieces of a cooked apple (M); mealworms or small pieces of cashew nut (S)		
	For a detailed description of the memory tasks see Schubiger et al. (2016)		
