# Supplementary material for: Does opportunistic testing bias cognitive performance in primates? Learning from drop-outs
Source: PLoS One. 2019 Mar 20;14(3):e0213727. doi: 10.1371/journal.pone.0213727 (PMC6426242; doi:10.1371/journal.pone.0213727)
Supplement: S1 Fig — Successful detour-reaching in the inhibition trials at (a) first attempt and (b) after initial failure to reach around the transparent barrier. Marmosets (grey solid bars)/squirrel monkeys (yellow dotted bars). (PDF) [file pone.0213727.s002.pdf]

a)

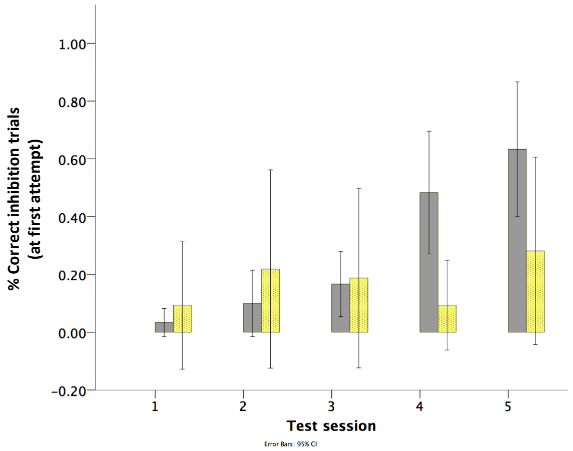

b)

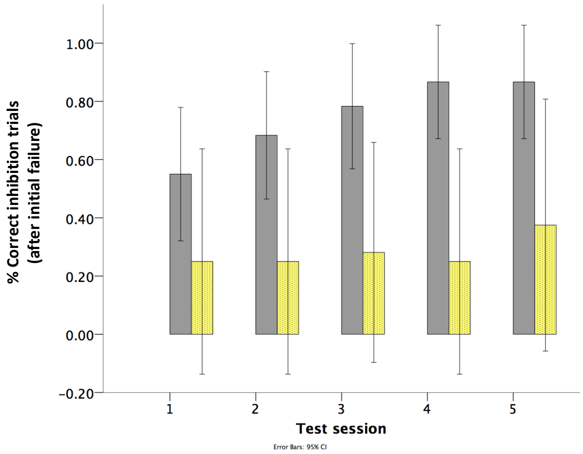

S1 Fig. Performance in the 5 test sessions of the Detour-Reaching task. Successful detour-reaching in the inhibition trials at (a) first attempt and (b) after initial failure to reach around the transparent barrier. Marmosets (grey solid bars)/squirrel monkeys (yellow dotted bars).
