# Supplementary material for: Does opportunistic testing bias cognitive performance in primates? Learning from drop-outs
Source: PLoS One. 2019 Mar 20;14(3):e0213727. doi: 10.1371/journal.pone.0213727 (PMC6426242; doi:10.1371/journal.pone.0213727)
Supplement: S2 Fig — Fewer (12-trial) sessions indicate better performance, hence the negative prefix on the y-axis. Phase 1—learning of the initial association between a pattern and a reward. Phase 2—strengthening of the learned association. Phase 3—reversal of the learned association. (PDF) [file pone.0213727.s003.pdf]

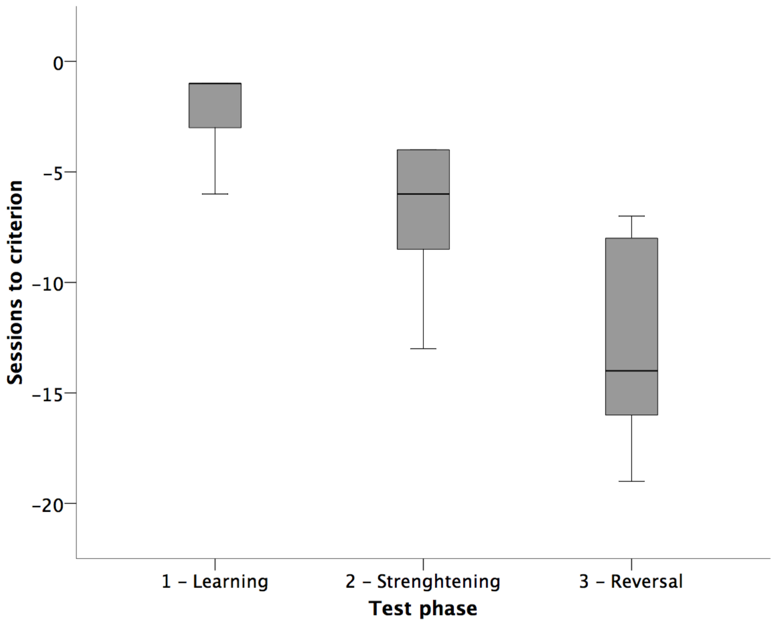

S2 Fig. Number of test sessions to criterion in the 3 phases of the Reversal Learning task. Fewer (12-trial) sessions indicate better performance, hence the negative prefix on the y-axis. Phase 1 - learning of the initial association between a pattern and a reward. Phase 2 - strengthening of the learned association. Phase 3 - reversal of the learned association.
