# Supplementary material for: Does opportunistic testing bias cognitive performance in primates? Learning from drop-outs
Source: PLoS One. 2019 Mar 20;14(3):e0213727. doi: 10.1371/journal.pone.0213727 (PMC6426242; doi:10.1371/journal.pone.0213727)
Supplement: S3 Fig — The dotted line represents the expected amount of time (number of testing days) after which testing would have been discontinued and subjects who needed longer would have been excluded had we used the classical opportunistic approach. (a) The marmosets of study sample 1 (represented by ID numbers M1-M16 and gender symbols) were tested with version a of Memory 1 (adapted from Banerjee et al., 2009), (b) the marmosets of study sample 2 (ID numbers M17-M27 and gender symbols) and the squirrel monkeys (represented by yellow triangles) were tested with version b (Schubiger et al., 2016). The two versions of Memory 1 slightly differed in details of experimental set-up (apparatus) and design (number of delay conditions; also see Fig 1 and S1 Table). (PDF) [file pone.0213727.s004.pdf]

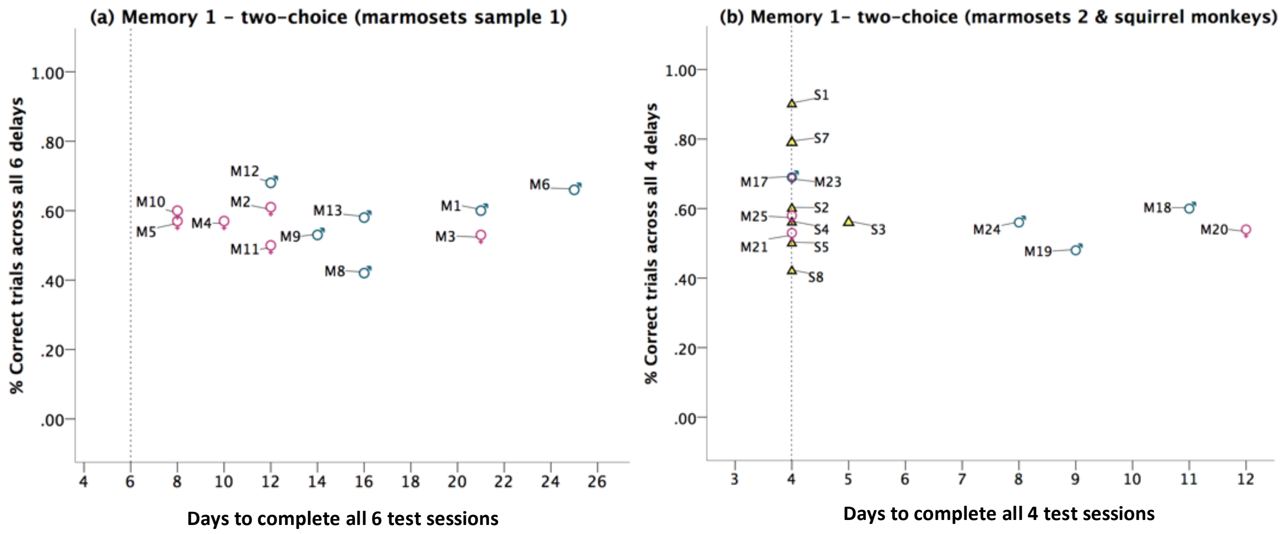

S3 Fig. The subjects' performance in the two versions of Memory task 1 depending on how much testing time they needed to complete the task. The dotted line represents the expected amount of time (number of testing days) after which testing would have been discontinued and subjects who needed longer would have been excluded had we used the classical opportunistic approach. (a) The marmosets of study sample 1 (represented by ID numbers M1-M16 and gender symbols) were tested with version a of Memory 1 (adapted from Banerjee et al., 2009), (b) the marmosets of study sample 2 (ID numbers M17-M27 and gender symbols) and the squirrel monkeys (represented by yellow triangles) were tested with version b (Schubiger et al., 2016). The two versions of Memory 1 slightly differed in details of experimental set-up (apparatus) and design (number of delay conditions; also see Fig 1 and S1 Table).
